# Combined Influences of Dementia Exposure and Personality on Self-Reported Memory Problems

**DOI:** 10.1177/1533317519899792

**Published:** 2020-03-12

**Authors:** Jacqueline Mogle, Nikki L. Hill, Tyler Reed Bell, Sakshi Bhargava, Emily Bratlee-Whitaker, Rachel K. Wion, Pooja Anushka Tiwari

**Affiliations:** 1 Edna Bennett Pierce Prevention Research Center, Penn State University, State College, PA, USA; 2 College of Nursing, Pennsylvania State University, State College, PA, USA; 3 Canyonville, OR, USA

**Keywords:** dementia, first-degree relatives, subjective memory, personality, self-reported memory

## Abstract

The current study investigated whether having a first-degree relative with dementia influenced older adults’ self-reported memory, if personality traits moderated these associations, and whether these associations differed by the type of item asked (ie, frequency of memory problems vs perceived memory decline). Data drawn from the Einstein Aging study included 454 older adults (*M*_age_ = 76.64, standard deviation = 4.77, 66.96% white, and 63% female). Multilevel modeling analyses showed participants who had a first-degree relative with dementia reported more frequent memory problems and were more likely to report memory decline over the past year. Among participants with a first-degree relative with dementia, higher levels of neuroticism were related to reports of more frequent memory problems at baseline, whereas higher levels of conscientiousness and lower levels of extraversion were related to reports of more frequent memory problems over time. Future research should consider personality traits and family history of dementia as potential contributors to self-reported memory problems.

## Introduction

Self-reported memory (SRM) problems are constructed judgements of memory functioning^1^ considered important in identifying individuals who are likely to develop Alzheimer disease and related dementias. Older adults with normal cognitive performance who report memory problems are over twice as likely to develop dementia when compared to those without SRM problems.^2,3^ However, other factors (eg, personality and dementia in first-degree relatives [FDRs]) can also influence memory problem reports. If a close relative has a diagnosis of dementia, an individual may ascribe different meanings to their own memory problems, altering judgements and perceptions of their memory.^4^ Identifying closely with someone who is currently experiencing, or has previously experienced, cognitive impairment or dementia may lead to reevaluation of an individual’s own memory problems^4^ and alter their reports in clinical and research settings; this may be intensified for people who are sensitive to threatening situations (eg, individuals higher in neuroticism). Exploring this possibility, the current study examines how having an FDR with dementia impacts older adults’ SRM, change in SRM over time, and whether this is moderated by personality traits such as neuroticism.

Previous work suggests up to 30% of older adults are concerned about developing memory problems and eventual dementia,^5,6^ and this concern may be higher for individuals with an FDR with dementia.^5,7^ For example, individuals with a parental history of Alzheimer disease are more likely to engage in “symptom-seeking” behavior, including repeatedly checking for signs of the disease, interpreting memory problems as indicative symptoms, and seeking validation from health-care providers.^8^ However, the impact of dementia in FDRs on older adults’ SRM requires greater clarification. For example, Heun and colleagues^9^ found no difference in the prevalence of memory complaints among older adults who had FDRs with Alzheimer disease (n = 718), spouses with Alzheimer disease (n = 146), or no relatives or spouses with Alzheimer disease (n = 136). In contrast, Tsai et al^10^ found that older adults with FDRs with Alzheimer disease (n = 1203) were almost twice as likely to report memory complaints than older adults with spouses having Alzheimer disease (n = 296; odds ratio [OR] =1.9, 95% confidence interval [CI] = 1.3-3.0). These results highlight the inconsistencies in our current understanding of how having an FDR with dementia impacts an individual’s perceptions of their memory functioning. Whether a patient has FDRs with dementia may be particularly important for clinicians to consider when evaluating memory complaints in cognitively healthy older adults.

A potential modifier of this relationship that may contribute to the inconsistent results above, but has not been included in previous examination, is personality. Personality, as defined by the Five Factor Model^11^ (FFM; neuroticism, extraversion, openness, agreeableness, conscientiousness), is known to influence reports of memory problems as well as general health concerns.^12^ Indeed, higher neuroticism, lower conscientiousness, and lower extraversion have all been linked to greater reports of memory problems.^13-16^ Different processes are hypothesized for each of these traits. Individuals higher in neuroticism tend to experience more intrusive thoughts, reducing attentional control and cognitive performance^17,18^ and tend to be more sensitive and likely to report somatic symptoms.^19^ Individuals with higher levels of neuroticism may therefore perceive memory problem as a potential indicator of dementia and report more memory problems compared to individuals with lower levels of neuroticism.^14^ Individuals with higher levels of conscientiousness tend to have higher self-control and be more hard working and organized^20^ compared to those lower in conscientiousness. They may be more likely to monitor their memory ability accurately and engage in strategies that help reduce memory lapses,^21,22^ leading them to experience and report fewer memory problems than individuals lower in conscientiousness. Finally, individuals higher in extraversion are more sociable, more optimistic, and are less likely to report health problems in general.^23^ In line with this tendency, individuals higher in extraversion are less likely to report memory problems compared to individuals lower in extraversion.^14^ Additionally, individuals higher in extraversion tend to perceive their memory as better than most^15^ and have more confidence in their judgments about memory performance,^24^ suggesting a more positive outlook on their memory functioning compared to those with lower extraversion.

Personality could then modify the experience of having an FDR with dementia in important ways. Individuals who are already predisposed to concerns about their health (ie, individuals higher in neuroticism) may have these concerns exacerbated by the discovery that a close relative has dementia and the perceived implications for their own susceptibility to this outcome. We would therefore expect that if having an FDR relates to judgements of poorer memory performance, then individuals higher in neuroticism would report greater impacts compared to individuals lower in neuroticism. The ways in which extraversion and conscientiousness might modify the effect of having an FDR with dementia are less clear. Previous longitudinal studies demonstrate inverse associations between conscientiousness and extraversion with SRM problems,^14,25^ but their protective effect in the context of familial dementia is unknown. When finding out an FDR has dementia, such personality traits might push older adults to utilize adaptive coping responses (ie, planning and instrumental social support)^26^ that reduce dementia anxiety through acquired information about contributing lifestyle factors^27^ and motivation to engage in preventive behaviors.^28^ Nevertheless, conscientious persons highly value their analytical abilities and might respond more negatively about potential degradation and be more sensitive to memory lapses.^29^ We will explore these moderated relationships in the current study to determine the extent to which they are important for understanding self-reports of memory problems.

In addition to personality, the approach to the measurement of SRM problems may contribute to inconsistent findings in prior work. In their review of the literature, Rabin et al^30^ found little consistency in the type of SRM items across preclinical Alzheimer disease studies. Some items ask participants to rate their current memory functioning (ie, self-rated memory) or decide if their memory has grown worse over time (ie, perceived memory decline). Moreover, self-reports ask participants to reflect on memory performance across different time periods (eg, recently or several years prior). Different items might tap into distinct constructs of SRM and thus help explain divergent findings in the field. For example, Heun et al^9^ and Tsai et al^10^ both examined the role of Alzheimer disease in close relatives with reports of memory problems in cognitively intact older adults. While Tsai et al^10^ found a relationship between having an FDR with Alzheimer disease and greater SRM problems, Heun et al^9^ did not find this association. Conflicting findings could be related to the measurement of SRM: Heun et al^9^ measured perceived memory decline (*changes in memory ability compared to earlier periods of life*), while Tsai et al^10^ measured self-rated memory (*trouble remembering things from one second to the next*). These findings underline a limitation in the current work: the assumption that memory problems derive from a unitary construct. Specifically, people who report memory problems have similar experiences, even if their problems are qualitatively different (eg, feeling forgetful versus feeling like memory is declining). In response to these gaps, this study examined associations between dementia in FDRs, personality, and different types of memory items to better understand these relationships.

The purpose of this study was to examine the unique influence of dementia in FDRs on SRM among older adults without cognitive impairment and to consider how the type of item may influence these relationships (ie, frequency of memory problems and perceived 1- and 10-year memory decline). Furthermore, we examined how these relationships may be modified by the 3 personality traits previously found to relate to SRM problems (ie, neuroticism, conscientiousness, and extraversion) and change in SRM problems over time.

## Methods

### Participants

Data were drawn from the Einstein Aging Study, a longitudinal cohort study examining cognitive aging and dementia among community-dwelling older adults (70+ years) in an urban, multiethnic area of New York City. Participants completed detailed medical and neuropsychological examinations and surveys annually. The study protocol was approved by the Albert Einstein College of Medicine Institutional Review Board (see Katz et al^31^ for full study details). The current study included participants who completed items on SRM, personality, and dementia in FDRs and excluded individuals with a diagnosis of amnestic mild cognitive impairment (MCI), non-amnestic MCI, or dementia.^32^ Diagnosis of MCI was made based on the updated Petersen criteria,^33^ including objective cognitive deficits greater than 1.5 standard deviations (SDs) below age-corrected normative mean scores. Dementia was diagnosed based on the clinical criteria from the *Diagnostic and Statistical Manual of Mental Disorders*-*IV* ^34^ applied in clinical case conferences.^31^

**Figure 1.**
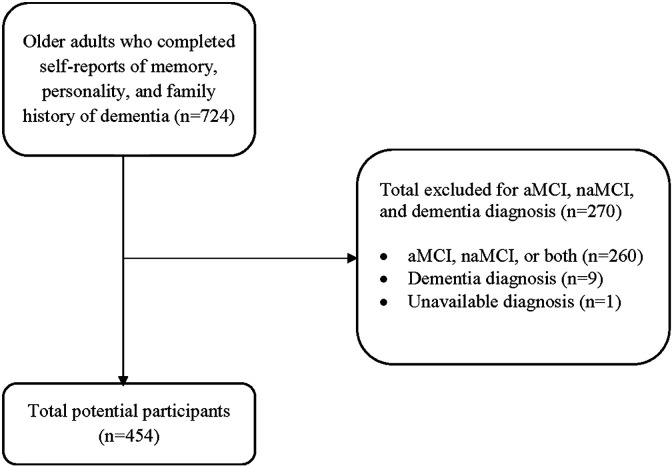
Sample selection diagram.

The current study included 454 participants (66.96% white, 25.77% black, 7.27% Other, and 63.0% female) at baseline who were at least 70 years old (*M*_age_ = 76.64, SD = 4.77) and had no clinical diagnosis of MCI or dementia at any point throughout the study period (See Figure 1 for more details). On average, participants completed 4 waves of data collection (*M*_waves_ = 3.98, SD = 2.68) with 10% of the sample completing 11 waves of data in the current study (n = 47). Consistent with other longitudinal studies with older adults, participants were lost to follow-up by about 15% to 20% at each wave, with later waves experiencing greater losses due to the age of the participants.^35,36^ Older adults with 4 or fewer waves of follow-up data did not differ from older adults with 5 or more waves of data on SRM or exposure to dementia in an FDR (*P* > .25); however, they did differ in their level of neuroticism (*t* [452] = 2.87, *P* = .004). Older adults with 4 or fewer waves of data had higher levels of neuroticism (*M* = 21.30, SD = 6.32) compared to older adults with 5 or more waves of data (*M* = 19.65, SD = 5.80). At baseline, participants had an average of 14.86 years of education (SD = 3.19). A total of 10.98% had an annual income below US$15 000 (ie, lived below poverty level); 32.46% of the participants’ annual income was between US$15 001 and US$30 000 (ie, lived at poverty level to up to 2 times above poverty level); and 56.56% of the participants had an annual income above US$30 000 (ie, lived more than 2 times above poverty level).

### Measures

#### Self-reported memory

Self-reported memory was assessed by 3 items at each wave. Frequency of memory problems was assessed with the item, “In the past year, how often did you have trouble remembering things?” with response options on a 4-point scale: 1 = never, 2 = rarely, 3 = sometimes, 4 = frequently. Perceived 1-year decline in memory was assessed with the item, “Compared with one year ago, do you have trouble remembering things more often, less often, or about the same?” Perceived 10-year decline in memory was assessed with the item, “Compared with ten years ago, do you have trouble remembering things more often, less often, or about the same?” Response options for these items included 1 = less often, 2 = more often, and 3 = about the same. These response options were recoded (0 = less often/about the same and 1 = more often), as only a few people indicated perceiving memory decline less often (2.13%-4.50%).

#### Personality

The 50-item International Personality Item Pool questionnaire^37^ was used to measure the FFM personality traits of which 3 personality traits were included in the current study: neuroticism (eg, “I often feel blue”), conscientiousness (eg, “I pay attention to details”), and extraversion (eg, “I feel comfortable around people”). Participants responded to 10 items for each personality trait, with response options ranging from 1 = very inaccurate to 5 = very accurate. Negatively worded items were reverse scored, and a total score was created for each personality trait, with higher scores indicating higher neuroticism, conscientiousness, and extraversion.

#### Dementia in FDRs

Participants were asked whether their father/mother/brother(s)/sister(s) had *severe memory impairment* (0 = No; 1 = Yes). This was coded as 1 (dementia in FDRs) and 0 (no dementia in FDRs). This general language provided greater inclusivity of relatives not yet formally diagnosed as well as relatives who have not yet disclosed official diagnosis.

#### Covariates

Participants’ age, sex (0 = female; 1 = male), race (1 = white; 2 = black; 3 = Other), income (1 = below US$15 000; 2 = between US$15 000 and US$30 000; 3 = more than US$30 000), and years of education were included as covariates.

### Statistical Analysis

Prior to examining the proposed research questions, descriptive analyses were performed to investigate if all variables of interest were normally distributed. Mean differences in age, education, income, frequency of memory problems, 1-year and 10-year memory decline, personality traits, and whether the participant had an FDR with dementia were examined by race and sex at baseline. Next, intercorrelations were examined among the key study variables. Correlations with categorical variables (eg, sex and race) were calculated using the Kendall Tau correction.

Multilevel modeling was performed in SAS (v. 9.4) to examine associations of older adults having an FDR with dementia with SRM (ie, frequency of memory problems, perceived 1-year memory decline, and perceived 10-year memory decline) at baseline and over time as well as whether personality traits moderated these associations. Multilevel modeling allows for the inclusion of individuals with different amounts of follow-up and computes estimates weighted for the amount of information each individual has contributed to different estimates. The simplest form of this equation (ie, without covariates) is:


1 $$\text{SR}{\text{M}}_{ij}={\text{ γ}}_{00}+{\text{ γ}}_{01}{\left(\text{FDR}\;\text{dementia}\right)}_{.j}+{\text{ γ}}_{02}{\left(\text{personality}\right)}_{.j}+{\text{ γ}}_{03}{\left(\text{FDR}\;\text{dementia}\;\times \;\text{personality}\right)}_{.j}+{\text{ γ}}_{10}{\left(\text{time}\right)}_{ij}+{\text{ γ}}_{20}{\left(\text{time}\;\times \;\text{FDR}\;\text{dementia}\right)}_{ij}+{\text{ γ}}_{30}{\left(\text{time}\;\times \;\text{personality}\right)}_{ij}+{\text{ γ}}_{40}{\left(\text{time}\;\times \;\text{FDR}\;\text{dementia}\;\times \;\text{personality}\right)}_{ij}+U_{0j}+{\text{ ∊}}_{ij}.$$

In Equation 1, γ_00_ represents the intercept of SRM across all individuals at baseline in the current study and *U_0j_* represents the random intercept variance allowing each person (*j*) to have a different intercept at baseline. γ_01_, γ_02_, and γ_03_ represent the effects of having an FDR with dementia, personality, and their interaction on SRM at baseline, respectively. γ_10_ represents the average change in SRM across time (*i*), while γ_20_, γ_30_, and γ_40_ represent the effect of having an FDR with dementia, personality, and their interaction on changes in SRM over time, respectively. Finally, ∊*_ij_* represents any residual error in the data after accounting for all other variables in the model.

Frequency of memory problems was treated as a continuous outcome and modeled using SAS PROC mixed. Perceived 1- and 10-year decline were binary variables (0 = less often/about the same and 1 = more often) and were modeled using SAS PROC GLIMMIX using a binary distribution with a logit link. First, empty models examined intraclass correlations to determine the proportion of variance in perceived frequency of memory problems and memory decline due to differences between individuals (relative to variance due to change across time). For the first set of substantive analyses, models examined the association of neuroticism, conscientiousness, extraversion, and having an FDR with dementia to SRM. Interaction of time with having an FDR with dementia was also included. Next, to examine whether the association of having an FDR with dementia with the 3 types of SRM significantly differed by older adults’ levels of neuroticism, extraversion, and conscientiousness, at baseline and over time, 2- and 3-level interactions were added to the model. Specifically, interactions of personality traits with time, personality traits with having an FDR, and personality traits with having an FDR and time were added in the model. Final models included only significant interaction terms. In case of a significant 3-way interaction, related lower level nonsignificant interactions were retained. For interactions involving continuous variables (eg, neuroticism), effects were estimated for individuals at ±1 SD from the mean to interpret effects and present results. Participants’ age, sex, race, education, and income were included as covariates in all models. Age and education were grand mean centered at baseline and income was dummy coded with the category US$15 001 to US$30 000 as the reference category. Additionally, personality variables and a dummy code for an FDR with dementia were included as between-person variables, and personality variables were grand-mean centered.

## Results

### Preliminary Analysis

Descriptive statistics of all key study variables are presented in Table 1. Statistics are provided for the entire sample and by whether or not individuals had an FDR with dementia. Intercorrelations among key study variables are presented in Table 2. See Supplemental Table 1 for descriptive statistics by race, sex, and income.

**Table 1.** Participant Characteristics.

Characteristics	Full Sample, N = 454	With Dementia FDR, n = 163	Without Dementia FDR, n = 291
Age, years, mean (SD)	76.64 (4.78)	76.58 (4.26)	76.68 (5.05)
Education, years, mean (SD)	14.86 (3.19)	14.73 (3.34)	14.94 (3.10)
Race, % (n)			
White	66.96 (304)	70.55 (115)	64.95 (189)
Black	25.77 (117)	24.54 (40)	26.46 (77)
Other	7.27 (33)	4.91 (8)	8.59 (25)
Sex, % (n)			
Male	63.22 (287)	63.80 (104)	62.89 (183)
Female	36.78 (167)	36.20 (59)	37.11 (108)
Income, % (n)			
<15K	10.98 (46)	6.71 (10)	13.33 (36)
15K-30K	32.46 (136)	34.23 (51)	31.48 (85)
>30K	56.56 (237)	59.06 (88)	55.19 (149)
Frequency of memory complaints, mean (SD)	2.64 (0.69)	2.76 (0.69)	2.57 (0.68)
One-year self-reported memory decline, mean (SD)	0.14 (0.35)	0.18 (0.39)	0.12 (0.33)
Ten-year self-reported memory decline, mean (SD)	0.61 (0.49)	0.64 (0.48)	0.59 (0.49)
Neuroticism, mean (SD)	20.54 (6.14)	20.99 (6.25)	20.30 (6.08)
Conscientiousness, mean (SD)	38.52 (6.25)	37.20 (6.77)	39.25 (5.83)
Extraversion, mean (SD)	34.18 (6.37)	33.93 (6.37)	34.31 (6.37)
Agreeableness, mean (SD)	40.82 (5.06)	40.34 (5.53)	41.08 (4.76)
Openness, mean (SD)	37.33 (6.42)	37.07 (6.68)	37.48 (6.28)

Abbreviations: Dementia FDR, first-degree relative with dementia; SD, standard deviation.

**Table 2.** Intercorrelations Among Key Study Variables at Baseline.^a^

Variable	1	2	3	4	5	6	7	8	9	10
1. Age	–									
2. Education	−.06	–								
3. Income (cat.)	−.09	.25^b^	–							
4. Frequency	−.02	.02	−.04	–						
5. One-year decline (cat.)	−.07	.03	−.00	.23^b^	–					
6. Ten-year decline (cat.)	.02	.01	.03	.33^b^	.29^b^	–				
7. Dementia in first-degree relatives (cat.)	−.01	−.03	.07	.13^c^	.08	.04	–			
8. Neuroticism	.05	−.12^b^	−.09	.13^b^	.18^b^	.12^c^	.05	–		
9. Conscientiousness	−.03	.09^c^	.05	−.12^b^	−.13^c^	−.06	−.16^b^	−.28^b^	–	
10. Extraversion	−.03	.08^d^	.05	−.09^d^	−.22^b^	−.13^c^	−.03	−.19^b^	.38^b^	–

Abbreviations: Cat., categorical variable; Frequency, frequency of self-reported memory problems.

^a^ Means and standard deviations only shown for continuous variables. Kendall Tau coefficients used for associations with categorical variables, Pearson used for continuous outcomes.

^b^ *P* < .001.

^c^ *P* < .01.

^d^ *P* < .05.

### Substantive Analysis

Intraclass correlation coefficients (ICCs) from the unconditional means model suggested that 48.95% of the variation in frequency of memory problems, 49.92% of the variation in perceived 1-year decline, and 57.37% of the variation in perceived 10-year decline are due to differences between participants. Below, we present findings for the associations of personality and family history with frequency of memory problems and perceived 1- and 10-year memory decline (see Table 3 for all coefficients).

**Table 3.** Association of Personality and Dementia in First-Degree Relatives with Self-Reported Memory Problems.^a^

	Frequency	1-Year Decline	10-Year Decline
	b (SE)	OR (95% CI)	OR (95% CI)
Intercept	2.59^b^ (0.07)	–	–
Time	0.01 (0.01)	1.12^c^ (1.04-1.21)	1.11^c^ (1.03-1.20)
Sex (ref = male)	0.11^d^ (0.06)	1.04 (0.63-1.71)	1.67 (0.98-2.83)
Education	0.01 (0.01)	1.07 (0.99-1.15)	1.00 (0.92-1.08)
Age	−0.01 (0.01)	0.94^d^ (0.89-0.99)	0.95 (0.90-1.00)
Black (ref = White)	−0.09 (0.06)	0.67 (0.37-1.21)	0.66 (0.36-1.21)
Other (ref = White)	0.10 (0.11)	0.95 (0.35-2.57)	0.61 (0.22-1.71)
Income > US$30 000 (ref = US$15 000-US$30 000)	−0.05 (0.06)	1.40 (0.81-2.42)	1.74 (0.99-3.05)
Income < US$15 000 (ref = US$15 000-US$30 000)	−0.05 (0.09)	1.24 (0.53-2.94)	0.93 (0.39-2.22)
Dementia FDR	0.11 (0.06)	2.41^c^ (1.36-4.25)	1.20 (0.68-2.14)
Neuroticism	0.00 (0.01)	1.05^d^ (1.01-1.10)	1.05 (1.00-1.10)
Conscientiousness	−0.01 (0.01)	0.98 (0.93-1.02)	0.99 (0.95-1.04)
Extraversion	−0.01^d^ (0.01)	0.94^c^ (0.91-0.98)	0.95^d^ (0.92-0.99)
Dementia FDR* Neuroticism	0.02^d^ (0.01)	–	–
Dementia FDR* Conscientiousness	0.003 (0.01)	–	–
Dementia FDR* Extraversion	0.02 (0.01)	–	–
Dementia FDR* Time	0.01 (0.01)	0.92 (0.82-1.02)	0.96 (0.86-1.07)
Conscientiousness* Time	−0.00 (0.001)	–	–
Extraversion* Time	0.00 (0.001)	–	–
Conscientiousness* Dementia FDR* Time	0.004^d^ (0.002)	–	–
Extraversion* Dementia FDR*Time	−0.004^d^ (0.002)	–	–

Abbreviations: CI, confidence interval; FDR, first-degree relative; Freq, frequency of memory problems; OR, odds ratio; SE, standard error.

^a^ Only significant interactions were retained in the models. In case of significant higher level interactions, related nonsignificant lower level interactions were also retained. Supplemental Table 2 includes these results with all 5 personality traits for completeness.

^b^ *P* < .001.

^c^ *P* < .01.

^d^ *P* < .05.

#### Frequency of memory problems

After accounting for the covariates, results showed that, on average, having an FDR with dementia was marginally related to frequency of memory problems (*b* = 0.11, standard error [SE] = 0.06, *P* = .06) at baseline and not significantly related over time (*b* = 0.01, SE = 0.01, *P* = .35). However, consistent with expectations, neuroticism moderated the association of having an FDR with dementia with frequency of memory problems (*b* = 0.02, SE = 0.01, *P* = .01) such that, on average, among older adults having an FDR with dementia, older adults with higher levels of neuroticism reported more frequent memory problems (*M* = 2.89, SE = 0.08, *P* < .001) compared to their counterparts (*M* = 2.57, SE = 0.08, *P* < .001). In contrast, among older adults without an FDR with dementia, frequency of memory problems was similar across levels of neuroticism (*M*_−1SD Neuroticism_ = 2.62, SE = .06; *M*_+1SD Neuroticism_ = 2.61, SE = .06).

**Figure 2.**
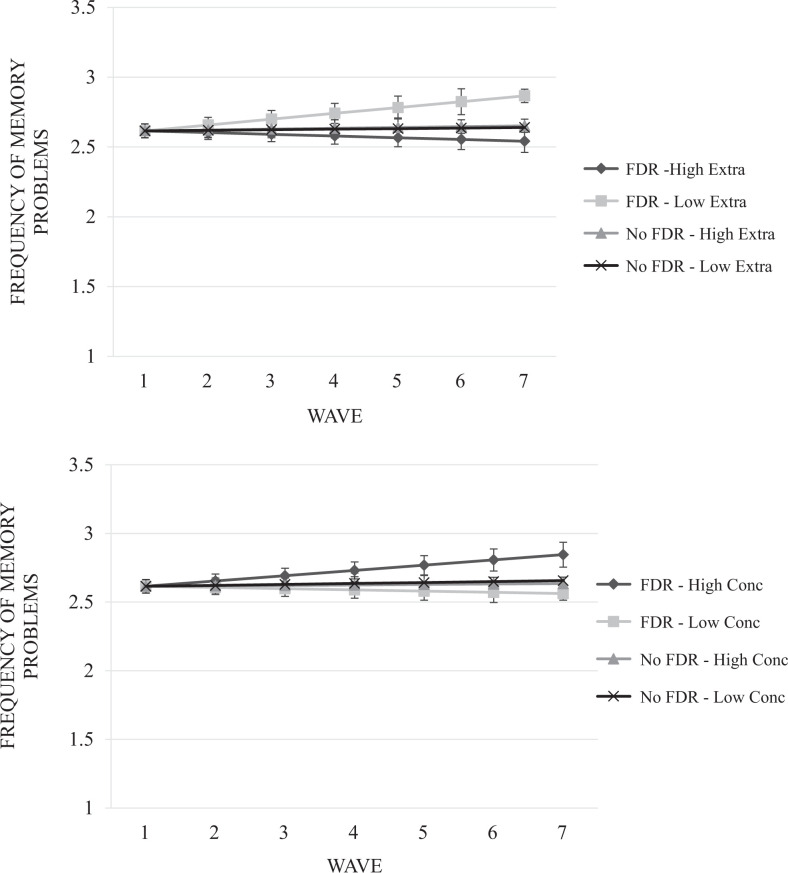
Extraversion and conscientiousness as the moderators of dementia in FDR and self-rated memory. *Note*. Top panel shows interaction of dementia FDR with extraversion, whereas the bottom shows the interaction of dementia FDR with conscientiousness. FDR indicates first-degree relative conscientiousness; Extra, Extraversion.

In our examination of change in frequency of memory problems across time, 2 significant interactions were uncovered (see Figure 2). First, a significant 3-way interaction was observed among conscientiousness, having an FDR with dementia, and time (*b* = 0.004, SE = 0.002, *P* = .02). Specifically, among older adults who had an FDR with dementia, those who also had higher levels of conscientiousness reported a greater frequency of memory problems over time (*b* = 0.04, SE = 0.01, *P* < .01), compared to those who had lower levels of conscientiousness (*b* = −0.01, SE = 0.01, *P* = .33). For older adults who did not have an FDR with dementia, their rate of change in frequency of memory problems did not differ by their level of conscientiousness (*P*s > .52). Additionally, a significant 3-way interaction was observed among extraversion, having an FDR, and time (*b* = −0.004, SE = 0.002, *P* = .01). Specifically, among older adults having an FDR with dementia, older adults with lower levels of extraversion reported greater frequency of memory problems over time (*b* = 0.04, SE = 0.01, *P* < .01) compared to older adults with higher levels of extraversion (*b* = −0.01, SE = 0.01, *P* = .25). For older adults with no FDR with dementia, frequency of memory problems changed at a similar rate over time irrespective of their level of extraversion (*Ps* > .53).

#### Perceived 1-year memory decline

After accounting for the covariates, results showed that, on average, older adults having an FDR with dementia were more likely to report a 1-year memory decline than their counterparts (OR = 2.41, 95% CI: 1.36-4.25). However, there was no relationship with changes in perceived 1-year memory decline over time (OR = 0.92, 95% CI: 0.82-1.02). Having an FDR with dementia did not interact with any of the personality traits, at baseline or over time, to predict perceived 1-year memory decline (*P*s > .12).

#### Perceived 10-year memory decline

Having an FDR with dementia was not related to perceived 10-year memory decline at baseline or over time (*P*s > .49). There were also no significant interaction effects (*P*s > .14).

## Discussion

The current study examined how dementia in FDRs impacted different types of SRM (frequency of problems vs perceived memory decline) in the context of personality traits. As suggested by some previous research, individuals who had an FDR with dementia were more likely to report a memory decline in the last year. However, having an FDR with dementia was not related to perceptions of decline over longer periods of time, suggesting that this effect is specific to recent evaluations of memory. Interestingly, the effect of having an FDR with dementia on the frequency of memory problems was exacerbated in individuals who were higher in neuroticism compared to those who were lower. This could indicate that the context of having a relative with dementia is interpreted differently by individuals depending on their level of neuroticism. Further, changes in reports of the frequency of memory problems over time were greater among individuals with lower extraversion or higher conscientiousness but only in the context of having a relative with dementia.

Our findings are consistent with some previous work suggesting that having a close relative with severe memory impairment impacts reports of memory problems (eg, Tsai et al^10^). Having exposure to someone with dementias like Alzheimer disease increases their knowledge about symptoms^5^ and, therefore, may make older adults more aware of their memory lapses and more sensitive to the potential implications of those problems. We did not find a relationship between having an FDR with dementia and perceptions of 10-year decline, which could be considered consistent with previous work by Heun and colleagues.^9^ Heun et al^9^ asked whether older adults perceived changes in memory ability compared to earlier periods in life. Using an indistinct temporal window increases the likelihood that older adults will vary in how they interpret the question, decreasing the validity of such items.^38^ Similarly, asking older adults to reflect on memory functioning over a specific time period (as in the current study with the past 10 years) increases the chances that they will rely on constructs other than actual memory performance, such as aging stereotypes, to provide a response. Importantly, this indicates that items asking about current memory functioning are more likely to be influenced by whether an older adult has an FDR with dementia, and this should be accounted for when considering reports of memory problems.

We also found that older adults higher in neuroticism who also had an FDR with dementia reported a higher frequency of memory problems compared to those lower in neuroticism. Findings suggest that while individuals higher in neuroticism are prone to higher anxiety about health in general,^39^ concerns about memory might require contextual cues. In particular, exposure to dementia in FDRs might convert general apprehension into specific anxiety about one’s memory. Aligning with constructed judgement hypothesis, such worries form negative beliefs about memory (ie, *memory worsens with age in my family*) which become self-affirmed through overinterpreting age-normative forgetting.^1^ Therefore, in older adults with exposure to dementia through a close relative, comprehensive measures of cognitive functioning (rather than brief cognitive screens) may be necessary to verify whether those higher in neuroticism are experiencing more forgetting or are simply more sensitive to instances of forgetting (cf., emotional reactions to daily stressors).^40^

Neuroticism is often implicated as a contributor to reports of memory problems in older adults, but our findings suggest a more complicated relationship. Conscientiousness and extraversion may also play important roles, depending on familial dementia exposure. We found that among participants having an FDR with dementia, those higher in conscientiousness or lower in extraversion tended to report more frequent memory problems over time. Previous studies report mixed results regarding conscientiousness, extraversion, and SRM,^14^ suggesting that other factors may be influencing these relationships. Higher conscientiousness is typically associated with better maintenance of cognitive performance throughout aging,^41,42^ similar to its associations with many positive aging-related outcomes such as lower comorbidity burden as well as mortality.^43^ Individuals higher in conscientiousness tend to engage in healthier behaviors, contributing to these improved outcomes.^44^ However, conscientious people are also detail- and goal-oriented, organized, and have a high need for achievement.^45^ Therefore, they may be more sensitive to subtle changes in cognitive performance that could influence their perceived ability to perform to expectations.^29^ Our results suggest that awareness of a close family member with dementia could heighten this sensitivity, leading to higher reports of memory problem frequency. Previous theorizing suggests that individuals higher in extraversion are more confident in their memory functioning and less likely to report memory problems.^24^ We found this association to be true only among older adults with a familial dementia history. Future examination of these relationships is necessary, as we did not find similar trends for conscientiousness and extraversion with the other SRM items.

There were limitations to the current study. First, the question about having an FDR with dementia focused on “severe memory impairment” rather than a formal diagnosis of Alzheimer disease or other dementia. Participants are using their judgment about whether their relative had “severe” impairment rather than having formal clinical indicators. Despite this, our findings are generalizable to situations, where individuals may have a relative with a memory disease that has not yet obtained clinical diagnoses suspected 61.7% of all cases^46^—and those that have not disclosed their official diagnosis. This general language also avoided problems caused by disparity, that is, many ethnoracial minorities and disadvantaged groups will be less likely to obtain formal diagnosis and thus less included in analyses of family history of dementia.^47^ Additionally, questions focused on parents and siblings, which assume a typical nuclear family structure. As family structures expand over time, considering other important family members that an individual is close to (eg, aunts, uncles, or grandparents) would enhance our understanding of how having a relative experiencing substantial changes in memory functioning impacts an individual’s rating of their own memory. This question also did not include spouses, but other studies consider this a unique group to be considered in the future.^9,10^ Importantly, the current sample did not include sufficient numbers of individuals in other race/ethnicity groups to understand how these processes might function in individuals with a Hispanic or Asian cultural background. Additionally, study attrition may have influenced our results. Although attrition in the current study was consistent with previous work in older adult samples,^35,36^ it is important to examine these relationships in larger samples with greater amounts of follow-up. We also did not include objective cognitive performance as a covariate. Future research should consider the best method of including objective cognitive performance in substantive models to account for changes in cognition over time. Finally, this study treated dementia in FDRs and personality as stable variables that do not vary over time. Future work should consider whether SRM changes *when* an individual finds out their relative has dementia. Similarly, some studies have identified personality changes with age^48^ that may impact older adults’ SRM over time as well.

Despite these limitations, this study has several strengths. The current sample had greater diversity (28% black) than previous work, making it more generalizable to the general population. We used 3 types of SRM to improve understanding of how aspects of the experience of perceived memory problems are influenced by individual characteristics, specifically personality and having a close relative with dementia. Additionally, although each of these was a single question, our analysis included reports over multiple years, improving measurement precision. Finally, the use of multilevel modeling allowed us to include all participants who had provided any data on our items of interest (regardless of the amount of follow-up), reducing the influence of survivor effects in the current analyses.

## Conclusion

The current study examined whether having an FDR with dementia or other severe memory impairment was related to older adults’ SRM and if this association varied by individuals’ personality traits. Results showed that older adults having an FDR with dementia were more likely to report memory decline in the last year. Additionally, older adults with higher levels of neuroticism and an FDR with dementia reported more frequent memory problems than older adults with higher levels of neuroticism but without an FDR with dementia. Further, older adults who had an FDR with dementia and had higher levels of conscientiousness or lower levels of extraversion reported an increase in frequency of memory problems over time. Future studies examining older adults’ SRM should account for their exposure to relatives with dementia as well as levels of neuroticism, conscientiousness, and extraversion, as these individual factors influence reports of problems in the absence of objective cognitive deficits.

## Supplemental Material

Supp2_Supplementary_Tables_12.03.2019 - Combined Influences of Dementia Exposure and Personality on Self-Reported Memory Problems Click here for additional data file. Supp2_Supplementary_Tables_12.03.2019 for Combined Influences of Dementia Exposure and Personality on Self-Reported Memory Problems by Jacqueline Mogle, Nikki L. Hill, Tyler Reed Bell, Sakshi Bhargava, Emily Bratlee-Whitaker, Rachel K. Wion and Pooja Anushka Tiwari in American Journal of Alzheimer's Disease & Other Dementias
