# Supplementary material for: Combined Influences of Dementia Exposure and Personality on Self-Reported Memory Problems
Source: Am J Alzheimers Dis Other Demen. 2020 Mar 12;35:1533317519899792. doi: 10.1177/1533317519899792 (PMC7135876; doi:10.1177/1533317519899792)
Supplement: Supp2_Supplementary_Tables_12.03.2019 - Combined Influences of Dementia Exposure and Personality on Self-Reported Memory Problems [file Supp2_Supplementary_Tables_12.03.2019.pdf]

Supplementary Tables

Table 1. *Baseline Mean Level Differences in Key Study Variables by Participants' Sex, Race, and Income.*

	**Total Sample** [%(n)]	**Frequency** [M(SD)]	**One-Year Decline (Yes)** [%(n)]	**Ten-year Decline (Yes)** [%(n)]	**Dementia in first-degree relatives** [%(n)]	**Neuroticism** [M(SD)]	**Conscientiousness** [M(SD)]	**Extraversion** [M(SD)]
**Sex differences**								
Female	63.2 (287)	2.72 (0.65)	14.34 (40)	63.08 (176)	36.24 (104)	21.08 (6.31)	38.46 (5.96)	33.92 (5.90)
Male	36.8 (167)	2.50 (0.73)	14.72 (24)	56.79 (92)	35.33 (59)	19.62 (5.74)	38.60 (6.74)	34.62 (7.09)
Significance Test		$t(439) = 3.36$	$\chi^2(1) = 0.01$	$\chi^2(1) = 1.70$	χ2(1) = .04	$t(452) = 2.45$	$t(452) = -0.23$	$t(298.74) = -1.12$
		$p = <.001$, $d = 0.32$	$p = .91$	$p = .19$	$p = .84$	$p = .01$, $d = 0.24$	$p = .82$, $d = 0.02$	$p = .26$, $d = 0.11$
**Race differences**								
White	67.0 (304)	2.67 (0.67)	14.24 (42)	63.39 (187)	37.83 (115)	20.80 (6.12)	38.74 (6.39)	34.07 (6.53)
Black	25.8 (117)	2.50 (0.76)	14.91 (17)	54.87 (62)	34.19 (40)	20.06 (6.28)	37.92 (6.31)	34.21 (6.10)
Other	7.3 (33)	2.82 (0.58)	15.15 (5)	57.58 (19)	24.24 (8)	19.91 (5.91)	38.54 (4.59)	35.00 (5.90)
Significance Test		$F(2,438) = 3.62$	$\chi^2(2) = 0.04$	$\chi^2(2) = 2.64$	χ2(2) = 2.59	$F(2,451) = 0.80$	$F(2,451) = 0.72$	$F(2,451) = 0.32$
		$p = .03$, $\eta^2 = .02$	$p = .98$	$p = .27$	$p = .27$	$p = .45$, $\eta^2 = .00$	$p = .49$, $\eta^2 = .00$	$p = .73$, $\eta^2 = .00$
**Income level differences**								
Below $15,000[1]	11.0 (46)	2.66 (0.74)	18.18 (8)	62.79 (27)	21.74 (10)	22.04 (6.02)	38.06 (5.51)	33.50 (5.80)
$15,000- $30,000[2]	32.4 (136)	2.67 (0.67)	12.03 (16)	55.64 (74)	37.50 (51)	20.76 (5.66)	38.23 (6.06)	34.02 (5.85)
>$30,000[3]	56.6 (237)	2.61 (0.70)	15.15 (35)	62.77 (145)	37.13 (88)	20.22 (6.55)	38.81 (6.64)	34.52 (6.83)

Significance Test	$F(2, 404) = 0.36$	$\chi^2(2) = 1.22$	$\chi^2(2) = 1.91$	$\chi^2(2) = 4.31$	$F(2, 416) = 1.73$	$F(2, 416) = 0.51$	$F(2, 416) = 0.61$
	$p = .70$, $\eta^2 = .00$	$p = .54$	$p = .38$	$p = .12$	$p = .18$, $\eta^2 = .01$	$p = .60$, $\eta^2 = .00$	$p = .54$, $\eta^2 = .00$

Note. Frequency = Frequency of self-reported memory problems.

Table 2. *Association of Five Personality Traits and Dementia in First-Degree Relatives with Self-Reported Memory*

	Frequency	One-Year Decline	Ten-Year Decline
	b (SE)	***OR (95%CI)***	***OR (95%CI)***
Intercept	2.58*** (0.07)	-	-
Time	0.005 (0.007)	1.12** (1.04 – 1.21)	1.11** (1.03 - 1.20)
Sex (ref = male)	0.12* (0.06)	1.03 (0.62 – 1.72)	1.56 (0.91 – 2.66)
Education	0.01 (0.01)	1.05 (0.96– 1.15)	0.99 (0.90 – 1.09)
Age	-0.01 (0.01)	0.94* (0.89 – 0.99)	0.95* (0.90 – 1.00)
Black (ref = White)	-0.19 (0.12)	0.70 (0.24 – 2.03)	1.07 (0.36 – 3.14)
Income > $30,000 (ref = $15,000 - $30,000)	-0.04 (0.06)	1.40 (0.81 – 2.42)	1.69 (0.97 – 2.97)
Income < $15,000 (ref = $15,000 - $30,000)	-0.05 (0.09)	1.26 (0.53 – 2.98)	0.91 (0.38 – 2.16)
Dementia FDR	0.11 (0.06)	2.42** (1.37 – 4.27)	1.23 (0.69 – 2.17)
Neuroticism	0.00 (0.01)	1.06* (1.01 – 1.11)	1.06* (1.01 – 1.12)
Conscientiousness	-0.01 (0.01)	0.97 (0.93 – 1.02)	0.99 (0.95 – 1.04)
Extraversion	-0.01* (0.01)	0.94** (0.90 – 0.98)	0.94* (0.90 – 0.99)
Agreeableness	-0.005 (0.01)	1.00 (0.95 – 1.06)	1.04 (0.98 – 1.10)
Openness	0.005 (0.005)	1.02 (0.97 – 1.06)	1.01 (0.97 – 1.06)
Dementia FDR* Neuroticism	0.02* (0.01)	-	-
Dementia FDR* Conscientiousness	0.002 (0.01)	-	-
Dementia FDR* Extraversion	0.02 (0.01)	-	-
Dementia FDR* Time	0.01 (0.01)	0.92 (0.82 – 1.02)	0.96 (0.86 – 1.07)
Conscientiousness* Time	-0.00 (0.00)	-	-
Extraversion* Time	0.00	-	-

	(0.001)		
Conscientiousness* Dementia FDR* Time	0.004* (0.002)	-	-
Extraversion* Dementia FDR*Time	-0.004* (0.002)	-	-

Note. Freq = Frequency of Memory Problems. FDR = First-Degree Relative. Only significant interactions were retained in the models. In case of significant higher level interactions, related non-significant lower level interactions were also retained. $^{***}p \leq .001$. $^{**}p \leq .01$, $^{*}p \leq .05$.
